# ﻿Description of two *Stenohya* species from China (Pseudoscorpiones, Neobisiidae), with comments on the exaggerated sexual dimorphic pedipalp in this genus

**DOI:** 10.3897/zookeys.1172.104773

**Published:** 2023-07-26

**Authors:** Nana Zhan, Zegang Feng, Xiangbo Guo, Feng Zhang

**Affiliations:** 1 The Key Laboratory of Zoological Systematics and Application, Institute of Life Science and Green Development, College of Life Sciences, Hebei University, Baoding, Hebei 071002, China Hebei University Baoding China; 2 Key Laboratory of Zoological Systematics and Evolution, Institute of Zoology, Chinese Academy of Sciences, Beijing 100101, China Institute of Zoology, Chinese Academy of Sciences Beijing China; 3 College of Life Sciences, University of Chinese Academy of Sciences, Beijing, 100049, China University of Chinese Academy of Sciences Beijing China

**Keywords:** Neobisiidae, new species, sexual dimorphism, taxonomy

## Abstract

Two *Stenohya* species belonging to the family Neobisiidae are diagnosed, described, and illustrated from China: *Stenohyaspinata***sp. nov**. from Chongqing Municipality, and *Stenohyahuangi* Hu & Zhang, 2012 from Fujian Province. The male of *S.huangi* is reported for the first time. Diagnostic characters of this species are restricted based on the holotype and the new specimens. In addition, a key and a distribution map of the *Stenohya* species from China are provided, and the potential function of the exaggerated sexual dimorphic pedipalp in the genus *Stenohya* is discussed.

## ﻿Introduction

The Asian pseudoscorpion genus *Stenohya* was erected and placed in Hyidae Chamberlin, 1930 by Beier (1967), with the type species *S.vietnamensis* Beier, 1967, whose holotype is a tritonymph. After reviewing some Asian species originally ascribed to the genus *Microcreagris* Balzan, 1892, Ćurčić (1983) erected the genus *Levigatocreagris*, with *Levigatocreagrisgruberi* Ćurčić, 1983 as the type species. However, Harvey (1991) regarded *Stenohya* as a senior synonym of *Levigatocreagris*, and transferred *Stenohya* to the family Neobisiidae Chamberlin, 1930 based on the presence of the venom apparatus only in the fixed chelal finger and a non-lanceolate trichobothrium *t*.

**Figure 1.**
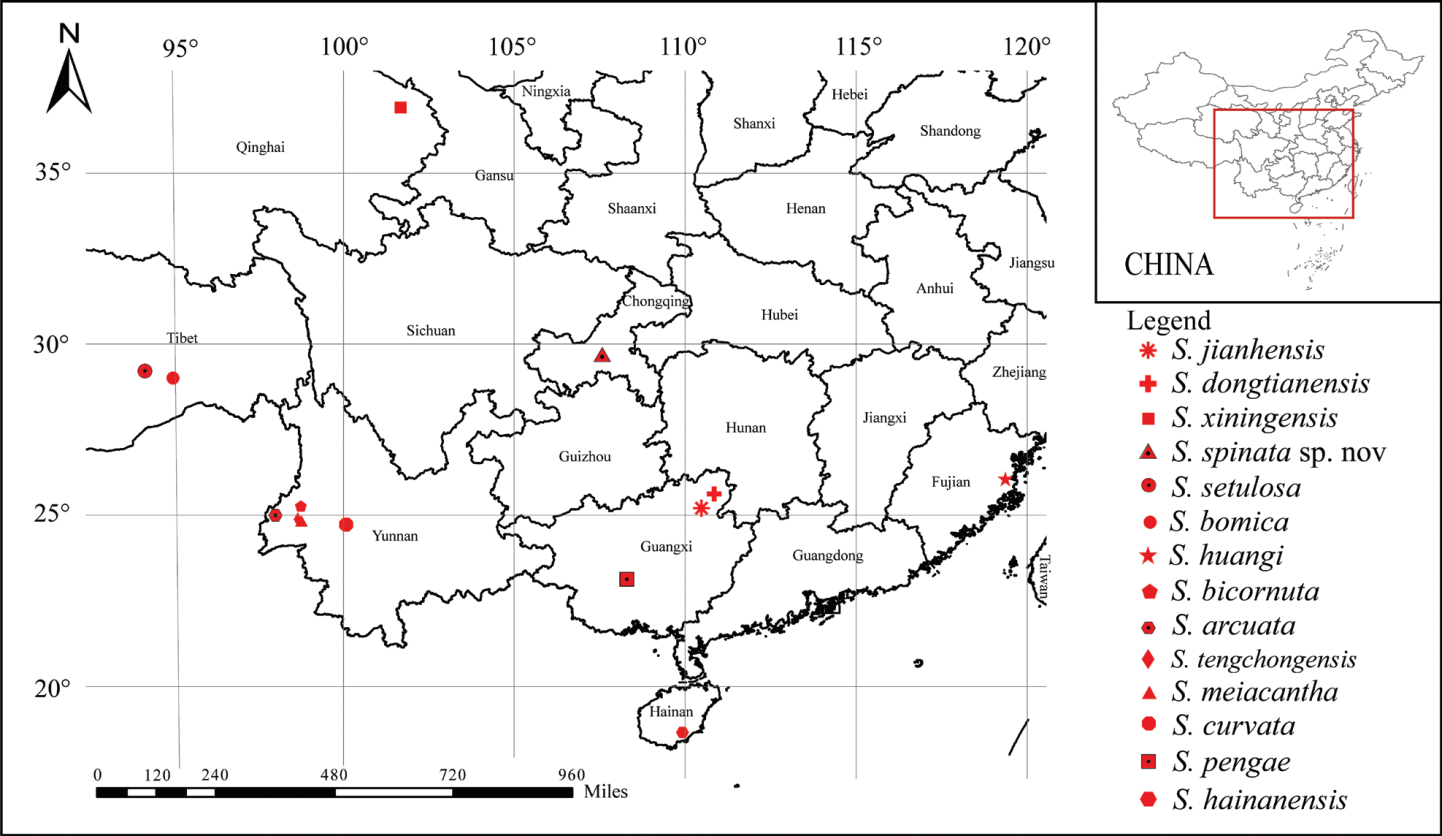
Distribution map of *Stenohya* species from China.

Until the present paper, this genus has contained 22 species distributed in Asia, with 13 of these described from China (Fig. 1): *S.curvata* Zhao, Zhang & Jia, 2011, *S.xiningensis* Zhao, Zhang & Jia, 2011, *S.bomica* Zhao & Zhang, 2011, *S.huangi* Hu & Zhang, 2012, *S.pengae* Hu & Zhang, 2012, *S.tengchongensis* Yang & Zhang, 2013, *S.meiacantha* Yang & Zhang, 2013, *S.hainanensis* Guo & Zhang, 2016, *S.setulosa* Guo & Zhang, 2016, *S.arcuata* Guo, Zang & Zhang, 2019, *S.bicornuta* Guo, Zang & Zhang, 2019, *S.dongtianensis* Li & Shi, 2023, and *S.jiahensis* Li & Shi, 2023 (Li and Shi 2023; WPC 2023). Most *Stenohya* live in leaf litter, with the exception of *S.pengae* which occurs in the canopy layer (Hu and Zhang 2012).

In this paper two *Stenohya* species with sexual dimorphic pedipalps are described and illustrated from China: *Stenohyaspinata* sp. nov. and *S.huangi*. Males of *S.spinata* sp. nov. have several strong thorns and spinous apophyses on the pedipalpal femur, patella and chela, whereas females lack these structures. The males of *S.huangi*, described here for the first time, have thinner pedipalps than females.

## ﻿Materials and methods

All specimens were examined, illustrated and measured using a Leica 205A stereomicroscope with a drawing tube. A detailed examination was carried out with an Olympus BX53 general optical microscope. Temporary slide mounts were prepared in glycerol. Figures were edited and formatted using Adobe Photoshop 2022. The specimens are preserved in 95% alcohol and deposited in the Museum of Hebei University (MHBU), Baoding, China. Terminology and mensuration largely follow Chamberlin (1931), except for the nomenclature of the pedipalps and legs, and the terminology of trichobothria (Harvey 1992); the term “rallum” (for flagellum) is adopted from Judson (2007).

The following abbreviations are used in the text for the trichobothria: ***b*** = basal; ***sb*** = sub-basal; **st** = subterminal; ***t*** = terminal; **ib** = interior basal; ***isb*** = interior subbasal; **ist** = interior subterminal; ***it*** = interior terminal; **eb** = exterior basal; ***esb*** = exterior subbasal; **est** = exterior subterminal; ***et*** = exterior terminal.

## ﻿Taxonomy

**Family Neobisiidae Chamberlin, 1930**

**Subfamily Microcreagrinae Balzan, 1892**

**Genus *Stenohya* Beier, 1967**

### ﻿ Stenohya spinata

Taxon classificationAnimaliaPseudoscorpionesNeobisiidae

Zhan, Feng & Zhang sp. nov.

A71E877A-7661-5C12-BF93-7627227D3C1F

https://zoobank.org/1F30AAF7-A979-4EA4-A802-60FB436310C0

**Figure 2.**
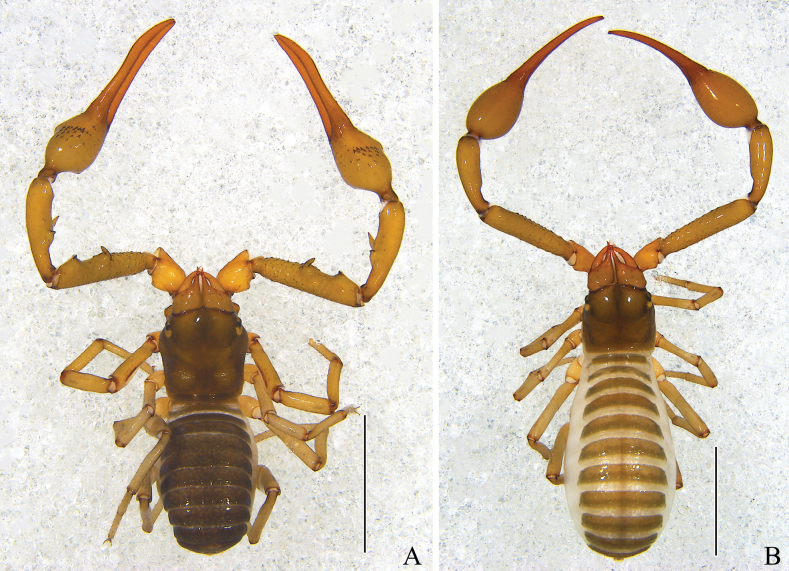
*Stenohyaspinata* sp. nov. **A** holotype male, dorsal view **B** paratype female, dorsal view. Scale bars: 2 mm.

**Figure 3.**
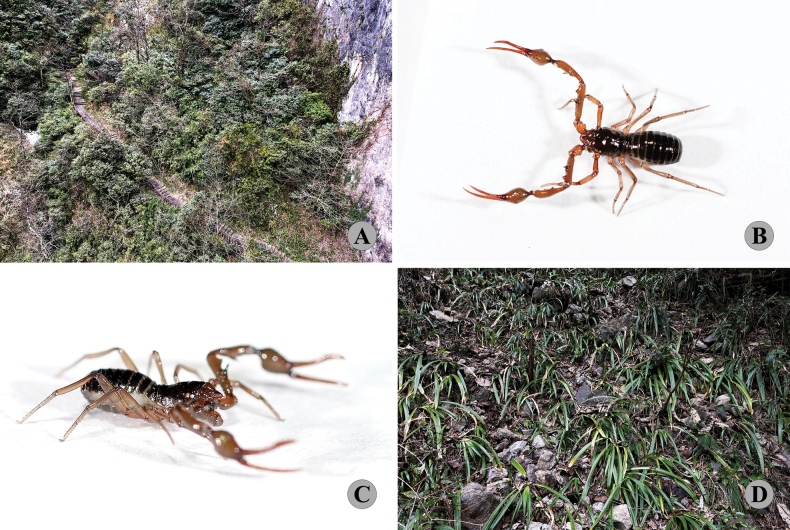
Type locality and habitus of *Stenohyaspinata* sp. nov. **A** vegetation surrounding the collection site in the Wuling Mountain Great Rift Valley **B, C** male habitus **D** litter layer inhabited by this species.

**Figure 4.**
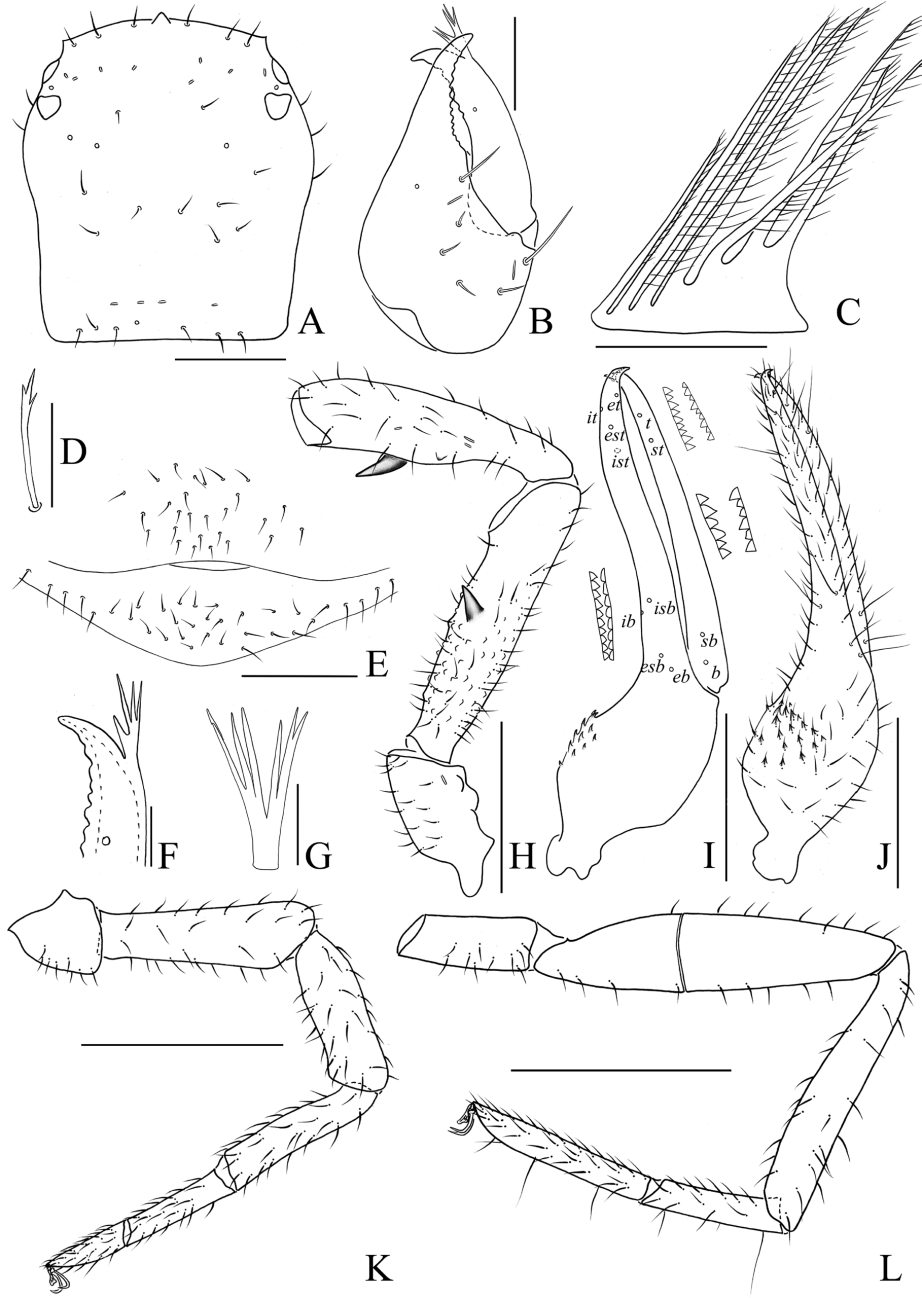
Holotype male of *Stenohyaspinata* sp. nov. **A** carapace, dorsal view **B** right chelicera, dorsal view **C** rallum **D** subterminal tarsal seta **E** chaetotaxy of genital area **F** top of movable cheliceral finger, showing galea **G** galea **H** right pedipalp, dorsal view (trochanter, femur, and patella) **I** right chela, lateral view, showing trichobothriotaxy, teeth and venom apparatus **J** right chela, dorsal view **K** right leg I, lateral view **L** right leg IV, lateral view. Scale bars: 1 mm (**H–L**); 0.5 mm (**A**); 0.25 mm (**B, E**); 0.1 mm (**C, D, F, G**).

**Figure 5.**
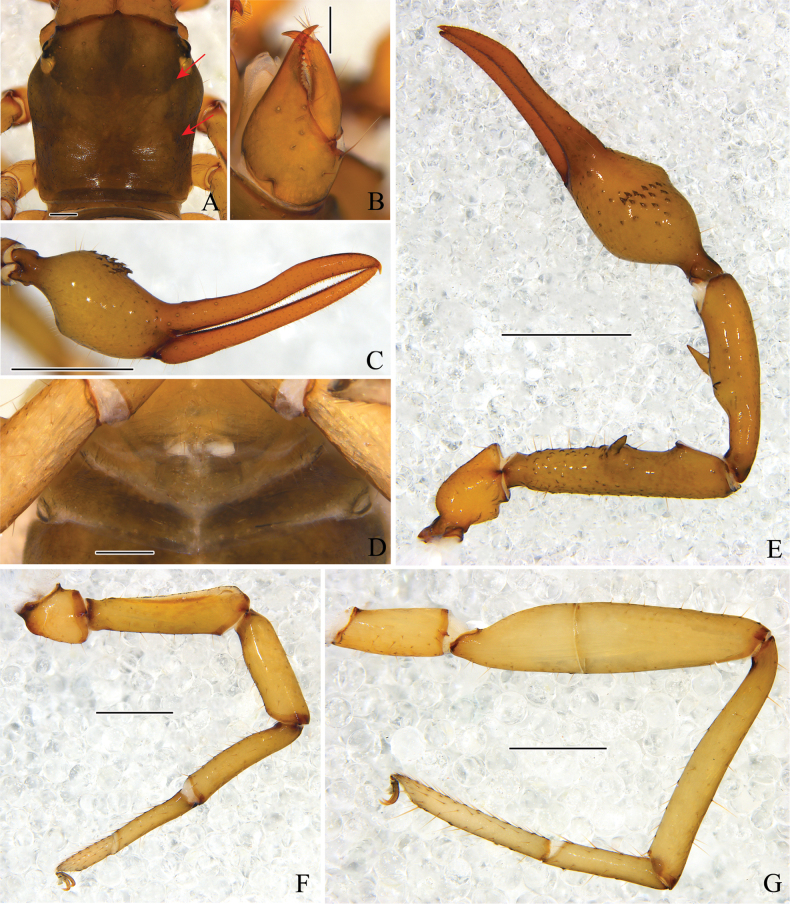
Holotype male of *Stenohyaspinata* sp. nov. **A** carapace, dorsal view (red arrows showing two transverse grooves) **B** right chelicera, dorsal view **C** right chela, lateral view **D** genital area **E** right pedipalp, dorsal view **F** right leg I, lateral view **G** right leg IV, lateral view. Scale bars: 1 mm (**C, E**); 0.5 mm (**F, G**); 0.2 mm (**A, B, D**).

**Figure 6.**
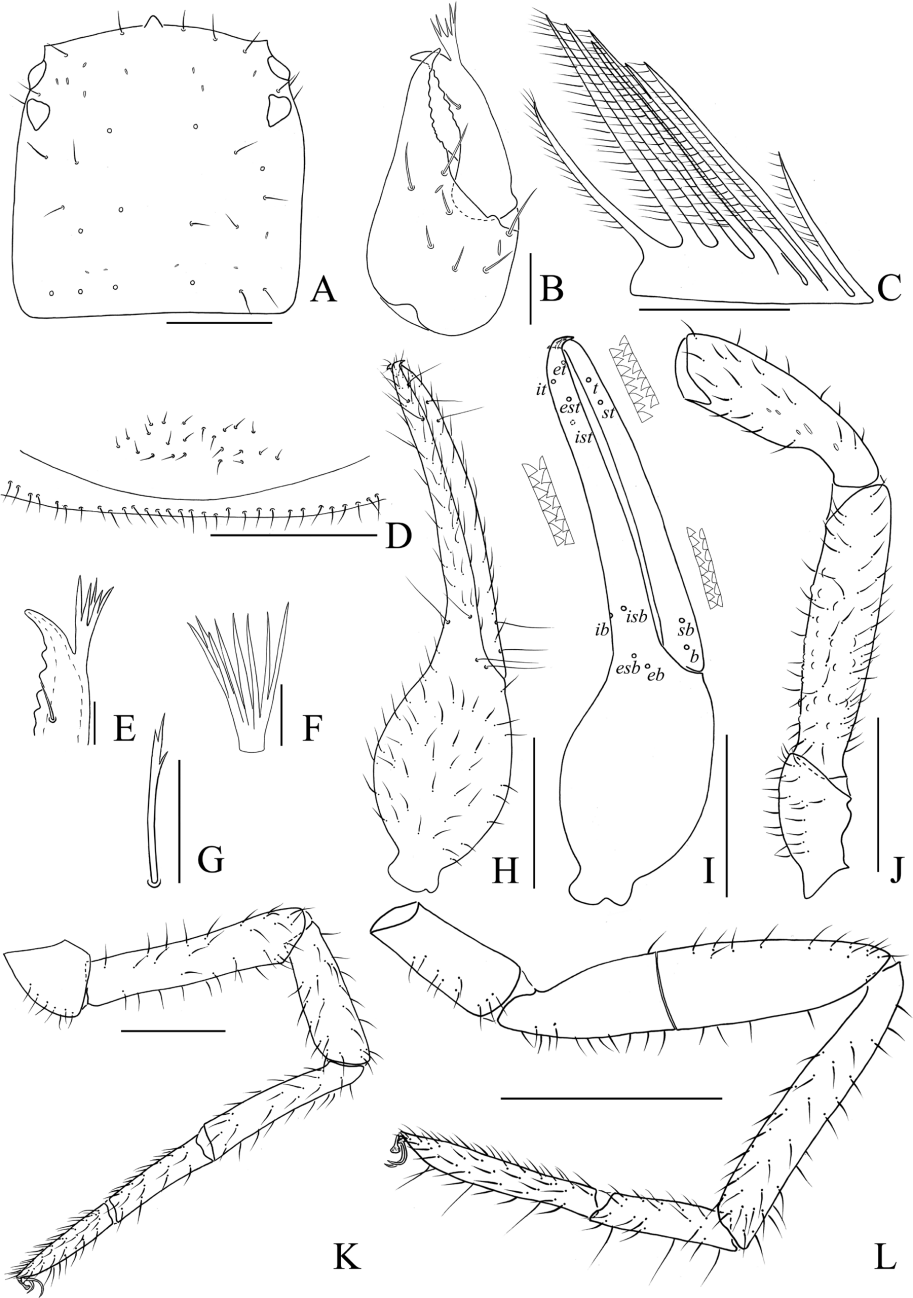
Paratype female of *Stenohyaspinata* sp. nov. **A** carapace, dorsal view **B** right chelicera, dorsal view **C** rallum **D** chaetotaxy of genital area **E** top of movable chelicera finger, showing galea **F** galea **G** subterminal tarsal seta **H** right pedipalp, dorsal view (trochanter, femur, and patella) **I** right chela, lateral view, showing trichobothriotaxy, teeth and venom apparatus **J** right chela, dorsal view **K** right leg I, lateral view **L** right leg IV, lateral view. Scale bars: 1 mm (**H–J, L**); 0.5 mm (**A, D, K**); 0.25 mm (**B**); 0.1 mm (**C, E–G**).

**Figure 7.**
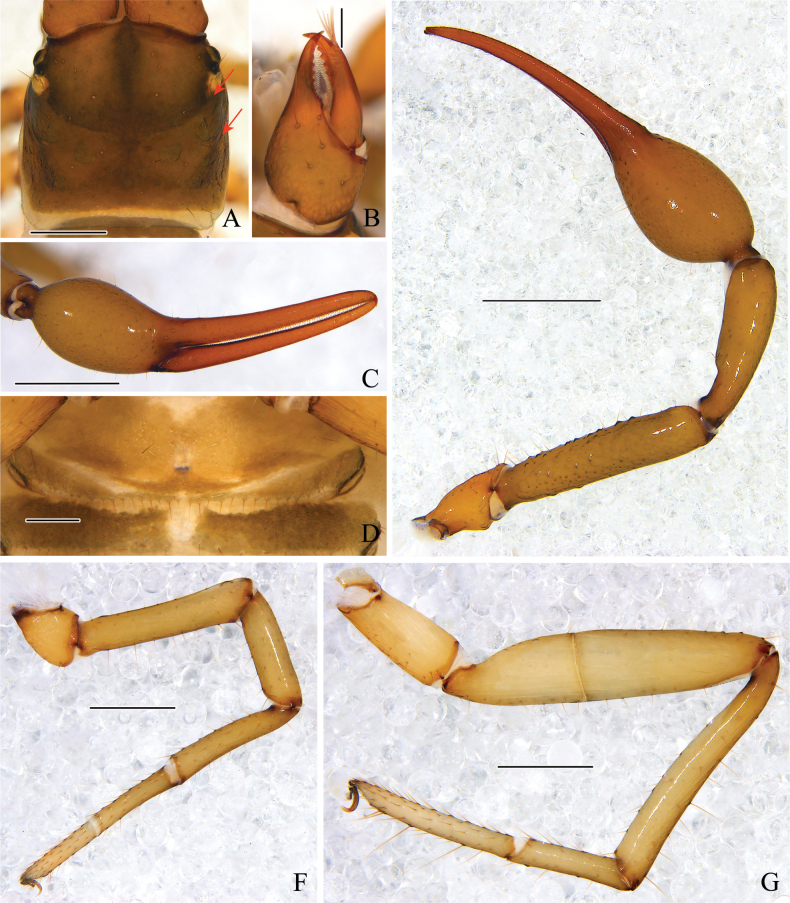
Paratype female of *Stenohyaspinata* sp. nov. **A** carapace, dorsal view (red arrows showing two transverse grooves) **B** right chelicera, dorsal view **C** right chela, lateral view **D** genital area **E** right pedipalp, dorsal view **F** right leg I, lateral view **G** right leg IV, lateral view. Scale bars: 1 mm (**C, E**); 0.5 mm (**A, F, G**); 0.2 mm (**B, D**).

Figs 2 , 3 , 4 , 5 , 6 , 7 Chinese name: 荆棘狭伪蝎

#### Type material

(Fig. 2). ***Holotype***: male (Ps.-**MHBU**-CQ2021120501), China: Chongqing Municipality, Fuling County, Wuling Mountain Great Rift Valley (Fig. 3A, D) [29°30′1.99″N, 107°34′50.15″E], alt. 1109m, 5 December 2021, collected from leaf litter and under rocks, Zhisheng Zhangand Luyu Wang leg. ***Paratypes***: 1 male (Ps.-**MHBU**-CQ2021120502), 3 females (Ps.-**MHBU**-CQ2021120503–05), same data as for holotype.

#### Etymology.

The specific name is derived from the Latin word “*spina*”, meaning thorn or spine, and refers to the spines on the pedipalpal femur, patella, and chelal hand.

#### Diagnosis.

Epistome triangular (Figs 4A, 5A, 6A, 7A); pedipalpal femur with many tubercles mainly surrounding the basal to median area (Figs 4H, 5E, 6J, 7E); male pedipalpal femur with a strong, long, peg-like thorn on the median prolateral position, as well as a projection on the subdistal prolateral surface (Figs 4H, 5E); male pedipalpal patella with a strong thorn medial-prolaterally, and a small projection near the base of this thorn (Figs 4H, 5E); male pedipalpal chela hand concaved at the ventral side of base, with 16 or 17 small, triangular, spinous apophyses on the medial-dorsal side, each spinous apophysis with a setae at the base (Figs 4I, J, 5C); male fixed chelal finger curved upward at median to distal part, movable chelal finger enlarged at base (Figs 4I, 5C).

#### Description.

**Adult male** (holotype and male paratypes) (Figs 2A, 3B, C).

***Carapace*** (Figs 4A, 5A). Carapace 1.07–1.08 times longer than broad, with a total of 29–33 setae, including 6 near anterior margin and 6–8 near posterior margin; with 4 pair lyrifissures near the anterior eyes. Carapace divided into 3 parts by 2 transverse, shallow grooves, the anterior part of uplift, the median part smooth, the posterior part with microgrooves; epistome big and triangular, with rounded apex; with 4 corneate eyes.

***Chelicera*** (Figs 4B, 5B). Hand with 7 setae and 2 lyrifissures, movable finger with 1 seta situated submedially; fixed finger with 14 teeth; movable finger with 7 or 8 teeth; serrula exterior with 41–46 lamellae; serrula interior with 34–38 lamellae; galea developed, 7 or 8 long branches divided from nearly the base (Fig. 4F, G); rallum consisting of 7 or 8 blades, all with anteriorly directed spinules, the basal-most blade shortest, base of the distal 3 or 4 blades thickened (Fig. 4C).

***Pedipalps*** (Figs 4H–J, 5C, E). Apex of pedipalpal coxa rounded, with 10 long setae. Femur with many big tubercles mainly surround the basal to median area, a strong, long, peg-like thorn on the median prolateral position, as well as a projection on the subdistal prolateral surface (Figs 4H, 5E); patella with a strong thorn on the median prolateral position, a small projection placed near the base of this thorn (Figs 4H, 5E); chelal hand concave at the ventral side of base, with 16 or 17 small, triangular, spinous apophyses on the median dorsal side, each spinous apophysis with a setae at the base (Figs 4I, J, 5E); fixed chelal finger curved upward at median to distal part; movable chelal finger enlarged at base (Figs 4I, 5C). Trochanter 1.63–2.02, femur 4.72–4.87, patella 4.30, chela (with pedicel) 4.07–4.11, chela (without pedicel) 3.68–3.73 times longer than broad, movable finger 1.46–1.47 times longer than hand (with pedicel). Fixed chelal finger with 8, movable chelal finger with 4 trichobothria: *eb*, *esb*, *ib*, and *isb* in basal fourth, *et*, *est*, *it*, and *ist* in distal fourth of fixed chelal finger; *b* and *sb* in basal fourth, *t* and *st* in distal fourth of movable chelal finger. Venom apparatus present only in fixed chelal finger, venom duct short (Fig. 4I). Fixed chelal finger with 96 or 97 cusped teeth, movable finger with 84–88 teeth, staggered arrangement of big and small teeth (Figs 4I, 5C).

***Abdomen***. Pleural membrane granulated. Tergites and sternites undivided, tergal chaetotaxy (I–XI): 6–7: 8: 7–8: 8–11: 11: 11–12: 11–13: 11–12: 11–12: 9–11: 10–11, sternal chaetotaxy (IV–XI): 23–26: 21–26: 28–30: 28–31: 27–30: 14–19: 12: 4, sternites VI–VIII with 11–13 medial scattered glandular setae, anal cone with 2 dorsal and 2 ventral setae. Genital area (Figs 4E, 5D): sternite II with total of 25–36 setae and 2 lyrifissures; sternite III with 21–23 setae and 2 lyrifissures anteriorly, 20 setae on posterior margin.

***Legs*** (Figs 4K, L, 5F, G). Legs generally typical, long and sinewy. Leg I: femur 3.21–3.31, patella 3.44–3.60, tibia 4.47–4.72, basitarsus 4.57–4.84, telotarsus 3.09–3.46 times longer than deep; femur 1.18–1.23 times longer than patella, telotarsus 0.54–0.59 times longer than basitarsus. Leg IV: femur + patella 4.61–4.79, tibia 7.05–7.15, basitarsus 4.60–4.79, telotarsus 7.17–7.42 times longer than deep; telotarsus 1.55–1.56 times longer than basitarsus; tibia with 2 sub-medial tactile setae (TS = 0.23, 0.65), basitarsus with 3 tactile setae (TS = 0.12–0.15, 0.42–0.45, 0.84–0.87), telotarsus with 2 tactile setae (TS = 0.23–0.35, 0.61–0.62); subterminal tarsal seta bifurcate (Fig. 4D). Arolium not divided, shorter than the slender and simple claws.

**Adult female** (paratype females) (Fig. 2B). Mostly same as males, except where noted.

***Carapace*** (Figs 6A, 7A). Carapace 1.07–1.11 times longer than broad, with a total of 28–31 setae, including 5 or 6 near anterior margin and 6–8 near posterior margin; carapace divided into 3 parts by 2 transverse, shallow grooves, with 6 pairs of lyrifissures near the anterior eyes.

***Chelicera*** (Figs 6B, 7B). Fixed finger with 11 or 12 teeth; movable finger with 6 or 7 teeth; serrula exterior with 46 or 47 lamellae; serrula interior with 40 lamellae; galea developed, 7 or 8 long branches divided from nearly the base (Fig. 6E, F); rallum consisting of 7 or 8 blades, all with anteriorly directed spinules, the basal-most blade shortest, base of the distal 3 or 4 blades thickened (Fig. 6C).

***Pedipalps*** (Figs 6H–J, 7C, E). Femur with many big tubercles mainly surround the basal to median area; chela smooth. Trochanter 1.83–2.16, femur 4.64–4.88, patella 3.53–3.62, chela (with pedicel) 4.02–4.10, chela (without pedicel) 3.78–3.84 times longer than broad, movable finger 0.57–0.64 times longer than hand (with pedicel). Fixed chelal finger with 82–91 cusped teeth, movable chelal finger with 76–78 cusped teeth.

***Abdomen.*** Tergal chaetotaxy (I–XI): 5–6: 10: 9: 10: 10–11: 12: 12: 12: 12: 9–11: 9–10, sternal chaetotaxy (IV–XI): 23–24: 21–23: 18–20: 19–21: 13–18: 14–16: 12: 4–5, sternites VI–VIII with 2 medial scattered glandular setae; genital area (Figs 6D, 7D): sternite II with total of 18–23 setae and 2 lyrifissures; sternite III with a row of 29–32 setae and 2 lyrifissures along posterior margin.

***Legs*** (Figs 6K, L, 7F, G). Leg I: femur 4.32–5.24, patella 3.44–3.62, tibia 5.00–5.46, basitarsus 4.31–4.70, telotarsus 4.10–5.10 times longer than deep; femur 1.19–1.52 times longer than patella, telotarsus 0.93–1.18 times longer than basitarsus. Leg IV: femur + patella 4.86–5.10, tibia 6.95–7.16, basitarsus 4.13–4.64, telotarsus 6.85–7.50 times longer than deep; telotarsus 1.34–1.38 times longer than basitarsus; tibia with 3 submedial tactile setae (TS = 0.22–0.25, 0.60–0.63, 0.94–0.96), basitarsus with 3 tactile setae (TS = 0.13–0.16, 0.45–0.47, 0.85–0.89), telotarsus with 2 tactile setae (TS = 0.28–0.29, 0.59–0.62); subterminal tarsal seta bifurcate (Fig. 6G).

#### Measurements

**(in mm; length/breadth or, for legs, length/depth).**

**Male** (holotype and paratypes). Body length 3.47–3.75. Carapace 1.42–1.46/1.31–1.36. Pedipalpal trochanter 0.89–0.90/0.44–0.49, femur 1.80–1.84/0.37–0.39, patella 1.65–1.67/0.37, chela (with pedicel) 3.01–3.04/0.74, chela (without pedicel) 2.72–2.76/0.74, hand length (without pedicel) 1.09–1.17, moveable finger length 1.67–2.02. Leg I: trochanter 0.49–0.50/0.34–0.35, femur 1.06/0.32–0.33, patella 0.86–0.90/0.25, tibia 0.85/0.18–0.19, basitarsus 0.63–0.64/0.13–0.14, telotarsus 0.34–0.38/0.11. Leg IV: trochanter 0.71/0.2–0.26, femur + patella 1.63–1.66/0.34–0.36, tibia 1.41–1.43/0.20, basitarsus 0.67–0.69/0.14–0.15, telotarsus 0.86–0.89/0.12.

**Female**. Body length 4.63–5.08. Carapace 1.19–1.33/1.10–1.28. Pedipalpal trochanter 0.77–0.97/0.42–0.45, femur 1.81–2.04/0.38–0.42, patella 1.41–1.52/0.39–0.41, chela (with pedicel) 3.08–3.48/0.77–0.85, chela (without pedicel) 2.91–3.26/0.77–0.85, hand length (without pedicel) 1.18–1.33, moveable finger length 1.75–2.22. Leg I: trochanter 0.42–0.48/0.29–0.33, femur 0.97–1.08/0.19–0.25, patella 0.65–0.76/0.19–0.21, tibia 0.74–0.84/0.14–0.16, basitarsus 0.50–0.56/0.11–0.13, telotarsus 0.47–0.51/0.10–0.11. Leg IV: trochanter 0.76–0.80/0.28–0.29, femur + patella 1.64–1.75/0.32–0.36, tibia 1.31–1.46/0.18–0.21, basitarsus 0.61–0.67/0.13–0.15, telotarsus 0.84–0.90/0.12.

#### Distribution.

China (Chongqing).

#### Remarks.

Like some other *Stenohya* species, *Stenohyaspinata* sp. nov. has exaggerated sexually dimorphic pedipalps, with those of the males armed with several strong thorns and spinous apophyses, which are absent in females. The presence of distinct apophyses on male pedipalps has been previously described in six *Stenohya* species: *S.hamata* (Leclerc & Mahnert, 1988), *S.curvata*, *S.meiacantha*, *S.bicornuta*, *S.dongtianensis*, and *S.jiahensis*. *Stenohyaspinata* can be distinguished from them by the position and shape of the projections on pedipalps, e.g. the chelal hand of males of *S.hamata* have a thorn-like projection on the ventral surface near the base of the fingers pointing distally downwards (Leclerc and Mahnert 1988); in *S.curvata*, males have a spine-like projection on the prolateral side of the chelal hand near the base of the fingers, they also have one or two small bulges between the projection and the finger (Zhao et al. 2011); in *S.meiacantha*, males have a spine-like projection on the prolateral side of the chelal hand near the base of the fingers (Yang and Zhang 2013); in *S.bicornuta*, the male chelal hand has a projection on the prolateral surface near the base of the fingers, and this projection with two horn-like bulges at the top (Guo et al. 2019); in *S.dongtianensis*, the male pedipalpal femur with one distal tubercle on the prolateral surface, chelal hand with 14 large tooth-shaped tubercles in the middle (retrolateral view) (Li and Shi 2023); in *S.jiahensis*, the male pedipalpal femur with one distal tubercle (many small bulges) and one basal tubercle on prolateral surface, chelal hand with 42 tooth-shaped tubercles (retrolateral view) (Li and Shi 2023); while in *S.spinata*, males have strong peg-like thorns on the median prolateral surface of the pedipalpal femur and patella, and have 16 or 17 spinous apophyses on the median dorsal side of chelal hand.

In the genus *Stenohya*, there are five species that lack descriptions of adult males: *S heros* (Beier, 1943) from Central Asia, *S.caelata* (Callaini, 1990) from India, and *S.bomica* from China were named and described from females, while *S.lindbergi* (Beier, 1959) from Afghanistan and *S.vietnamensis* from Vietnam were named and described from tritonymphs. *Stenohyaspinata* can be easily distinguished from them by having a triangular epistome (*S.vietnamensis* without epistome) (Beier 1967; Harvey 1991); pedipalpal femur with large tubercles (pedipalp smooth in *S.lindbergi* and *S.heros*) (Beier 1943, 1959; Ćurčić 1983); *ist* located at the distal fourth of the fixed chelal finger, distinctly closer to *it* (*ist* mostly halfway between *ib* and *it* in *S.caelata*) (Callaini 1990); *sb* located at the base of movable finger, close to *b* (*sb* located near the top of movable finger, close to *st* in *S.bomica*) (Zhao and Zhang 2011).

### ﻿ Stenohya huangi

Taxon classificationAnimaliaPseudoscorpionesNeobisiidae

Hu & Zhang, 2012

4AA74E3B-DCD0-5F0F-9873-EFBC31D6179A

**Figure 8.**
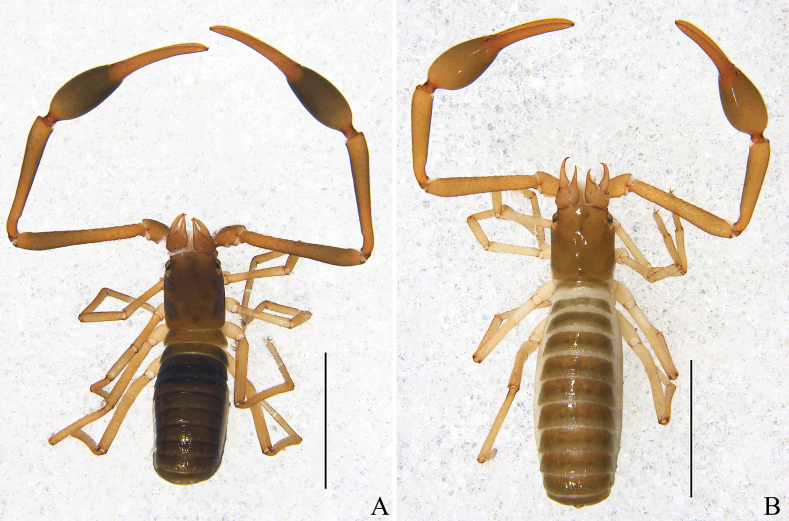
*Stenohyahuangi* Hu & Zhang, 2012 **A** male, dorsal view **B** female, dorsal view. Scale bars: 2 mm.

**Figure 9.**
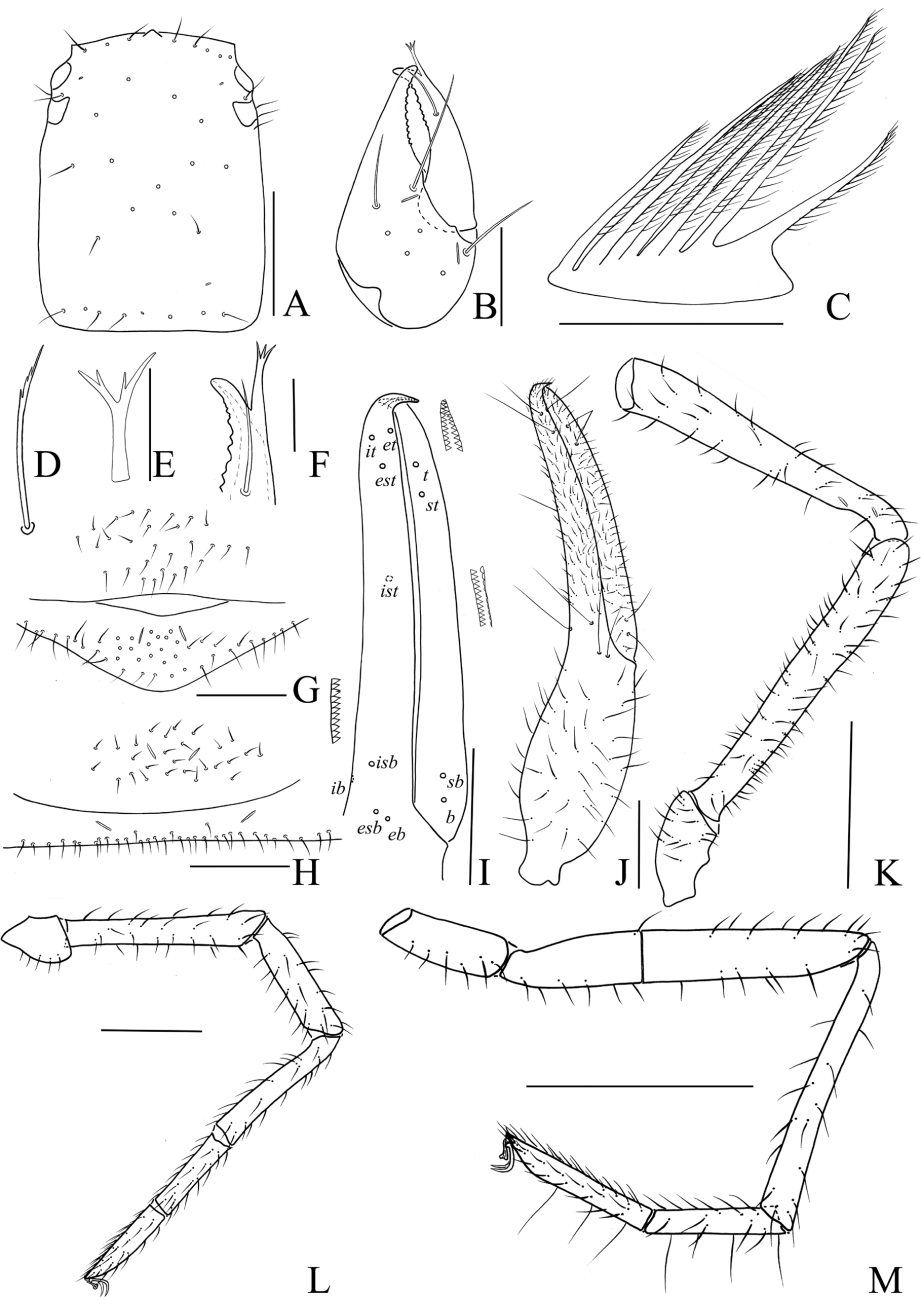
*Stenohyahuangi* Hu & Zhang, 2012, male (**A–G**, **I–M**), female (**H**) **A** carapace, dorsal view **B** right chelicera, dorsal view **C** rallum **D** subterminal tarsal seta **E** galea **F** top of movable cheliceral finger, showing galea **G** chaetotaxy of male genital area **H** chaetotaxy of female genital area **I** right chela, lateral view, showing trichobothriotaxy, teeth and venom apparatus **J** right chela, dorsal view **K** right pedipalp, dorsal view (trochanter, femur, and patella) **L** right leg I, lateral view **M** right leg IV, lateral view. Scale bars: 1 mm (**K**); 0.5 mm (**A, I, J, L, M**); 0.25 mm (**B, G, H**); 0.1 mm (**C, E, F**).

**Figure 10.**
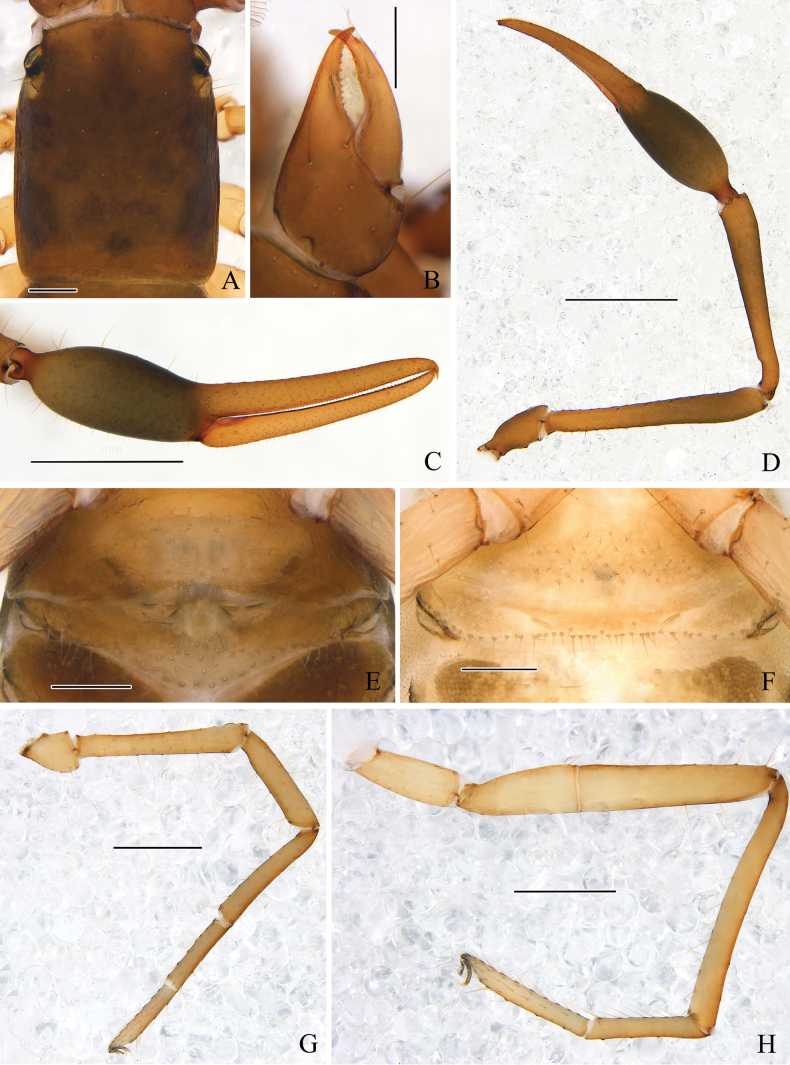
*Stenohyahuangi* Hu & Zhang, 2012, male (**A–E, G, H**), female (**F**) **A** carapace, dorsal view **B** right chelicera, dorsal view **C** right chela, lateral view **D** right pedipalp, dorsal view **E** male genital area **F** female genital area **G** right leg I, lateral view **H** right leg IV, lateral view. Scale bars: 1 mm (**C, D**); 0.5 mm (**G, H**); 0.2 mm (**A, B, E, F**).

**Figure 11.**
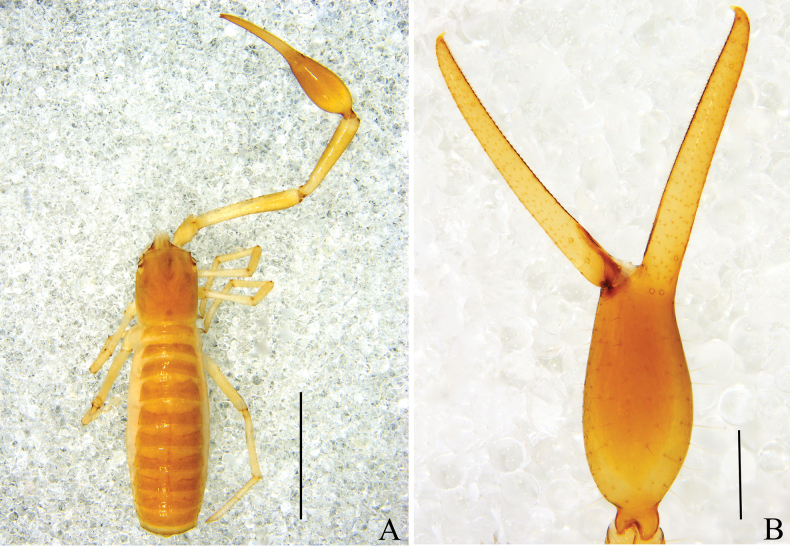
*Stenohyahuangi* Hu & Zhang, 2012, holotype female **A** dorsal view (chelicerae, left palp, left legI, leg IV and right leg III removed) **B** left chela, lateral view. Scale bars: 2mm (**A**); 0.5 mm (**B**).

Figs 8 , 9 , 10 , 11 Chinese name: 黄氏狭伪蝎

#### Material examined.

***Holotype*** female (Ps.-MHBU-FJ750224); 7 males (Ps.-MHBU-FJ2018040401–07), 4 females (Ps.-MHBU- FJ2018040408–11), China: Fujian Province, Fuzhou City, Gushan Mountain [26°5'39"N, 119°22'28"E], alt. 177m, 4 April 2018, collected from leaf litter, Xiangbo Guo, Weitong Wang and Xiao Zang leg.

#### Revised diagnosis.

Small body size; pedipalpal femur straight with tubercles on the median prolateral position (Figs 9K, 10D); male pedipalpal femur 7.64–7.96 (female 6.07–6.14), patella 6.32–6.45 (female 4.70–4.83), chela (with pedicel) 5.23–5.48 (female 4.26–4.56), chela (without pedicel) 4.98–5.19 (female 4.03–4.39) times longer than broad; male movable chelal finger with 30–33 (female 46–51) teeth situated at median to distal position (Figs 9I, 10C, 11B).

#### Description.

**Adult male**: (Fig. 8A).

***Carapace*** (Figs 9A, 10A). Smooth, carapace 1.45–1.55 times longer than broad, with a total of 30–34 setae, including 6–8 near anterior margin and 6–8 near posterior margin; 4 eyes, anterior pair with lens, posterior pair with weak lens; with 2 pair of lyrifissures near the anterior eyes; epistome small and triangular, with rounded apex.

***Chelicera*** (Figs 9B, 10B). Hand with 7 setae and 2 lyrifissures, movable finger with 1 seta situated submedially; fixed finger with 17–19 teeth; movable finger with 11–16 teeth; serrula exterior with 40–44 lamellae; serrula interior with 29 or 30 lamellae; galea elongated, divided into 2 main branches, 1 branch is secondarily divided into 3 terminal branches, while the other is divided into 2 or 3 branches (Fig. 9E, F); rallum consisting of 8 blades, all with anteriorly directed spinules, the basal-most blade shortest (Fig. 9C).

***Pedipalps*** (Figs 9I–K, 10C, D). Pedipalps long and slender (Fig. 10D). Apex of pedipalpal coxa rounded, with 4 long setae; femur straight, with tubercles on the median prolateral position, the other sections smooth; patella claviform, smooth; chelal fingers long and slender (Figs 9J, K, 10C, D). Trochanter 2.21–2.35, femur 7.64–7.96, patella 6.32–6.45, chela (with pedicel) 5.23–5.48, chela (without pedicel) 4.98–5.19 times longer than broad, movable finger 1.32–1.43 times longer than hand (with pedicel). Fixed chelal finger with 8, movable finger with 4 trichobothria: *eb*, *esb*, *ib*, and *isb* located basally of fixed chelal finger, *est*, *et*, and *it* situated distally of fixed chelal finger, *ist* situated midway between *isb* and *it*, nearer to *it* than to *isb*, *b* and *sb* closer to each other situated on the basal half, and *st* and *t* closer to each other situated on the distal half of the movable finger (Figs 9I, 10C); venom apparatus present only in fixed chelal finger, venom duct short (Fig. 9I); fixed finger with 71–77 pointed teeth, staggered arrangement of small and large teeth; movable finger with 30–33 teeth situated at median to distal position, rounded at median position, while pointed at distal position.

***Abdomen.*** Pleural membrane granulated. Tergites and sternites undivided, tergal chaetotaxy (I–XI): 4–7:8–10:10–11:10–11:12–13:11–14:11–17:11–14:11–12:10–11:10, sternal chaetotaxy (IV–XI): 26–27:23–28:32–39:38–42:30–39:16–22:16–18:6. sternites VI–VIII with 13–14 medial scattered glandular setae, sternites VI–X with 2 lyrifissures, anal cone with 2 dorsal and 2 ventral setae. Genital area (Figs 9G, 10E): sternite II with total of 26–29 setae and 2 lyrifissures; sternite III with a row of 47–51 setae and 2 lyrifissures.

***Legs*** (Figs 9L, M, 10G, H). Legs typical, long and sinewy. Leg I: femur 6.20–6.57, patella 4.27–4.64, tibia 5.50–5.75, basitarsus 4.90–5.11, telotarsus 5.33–5.56 times longer than deep; femur 1.42–1.52 times longer than patella, telotarsus 1.02–1.09 times longer than basitarsus. Leg IV: femur + patella 6.57–6.91, tibia 8.93–10.00, basitarsus 6.00–6.44, telotarsus 7.44–7.78 times longer than deep, telotarsus 1.16–1.19 times longer than basitarsus; basitarsus with 2 tactile setae (TS = 0.12–0.14, 0.86–0.87), telotarsus with 3 tactile setae (TS = 0.13–0.22, 0.29–0.39, 0.58–0.60); subterminal tarsal seta bifurcate (Fig. 9D). Arolium not divided, shorter than the slender and simple claws.

**Adult female** (Fig. 8B): Mostly same as males, except where noted.

***Carapace.*** Smooth, carapace 1.33–1.49 times longer than broad, with a total of 32–36 setae, including 7–9 near anterior margin and 7–9 near posterior margin; with 2 pair lyrifissures near the anterior eyes.

***Chelicera.*** Fixed finger with 23 teeth; movable finger with 11 teeth; serrula exterior with 36–39 lamellae; serrula interior with 35–39 lamellae; galea elongated and divided into 2 main branches, each branch secondarily divided into 3 terminal branches.

***Pedipalps.*** Trochanter 1.97–2.10, femur 6.07–6.14, patella 4.70–4.83, chela (with pedicel) 4.26–4.56, chela (without pedicel) 4.03–4.39 times longer than broad, movable finger 1.14–1.30 times longer than hand (with pedicel). Fixed finger with 63–69 pointed teeth; movable finger with 46–51 teeth situated at median to distal position, rounded at median position, while pointed at distal position.

***Abdomen.*** Tergal chaetotaxy (I–XI): 4–6:9–10:10–11:10:11–12:11–13:11–12:11–12:11:10–11:8–11, sternal chaetotaxy (IV–XI): 24–27:21–26:20–27:23–29:22–25:20–22:15–18:6. sternites VI–VIII with 2 medial scattered glandular setae. Genital area (Figs 9H, 10F): sternite II with total of 23–30 setae and 2 lyrifissures; sternite III with a row of 35–48 setae and 2 lyrifissures.

***Legs.*** Leg I: femur 5.93–6.21, patella 3.80–4.38, tibia 5.17–5.73, basitarsus 4.67–5.00, telotarsus 5.11–5.56 times longer than deep; femur 1.42–1.52 times longer than patella, telotarsus 1.09–1.11 times longer than basitarsus. Leg IV: femur + patella 5.18–6.46, tibia 8.40–8.86, basitarsus 5.18–5.50, telotarsus 6.50–7.10 times longer than deep; telotarsus 1.18–1.21 times longer than basitarsus; basitarsus with 2 tactile setae (TS = 0.13–0.16, 0.86–0.89), telotarsus with 3 tactile setae (TS = 0.14–0.19, 0.33–0.38, 0.56–0.58); subterminal tarsal seta bifurcate.

#### Measurements

**(in mm; length/breadth or, for legs, length/depth).**

**Male**. Body length 3.16–3.62. Carapace 1.10–1.17/0.73–0.80. Pedipalpal trochanter 0.61–0.67/0.26–0.30, femur 1.91–2.05/0.24–0.26, patella 1.77–1.87/0.28–0.29, chela (with pedicel) 2.52–2.77/0.46–0.53, chela (without pedicel) 2.38–2.64/0.46–0.53, hand length (without pedicel) 1.00–1.15, moveable finger length 1.43–1.52. Leg I: trochanter 0.32–0.35/0.21–0.22, femur 0.92–0.99/0.14–0.16, patella 0.64–0.65/0.14–0.15, tibia 0.66–0.74/0.12–0.13, basitarsus 0.45–0.49/0.09–0.10, telotarsus 0.48–0.50/0.09. Leg IV: trochanter 0.58–0.62/0.20–0.21, femur + patella 1.48–1.59/0.22–0.23, tibia 1.23–1.30/0.13–0.14, basitarsus 0.58–0.60/0.09–0.10, telotarsus 0.67–0.70/0.09.

**Female.** Body length 4.46–5.01. Carapace 1.16–1.19/0.80–0.89. Pedipalpal trochanter 0.59–0.61/0.29–0.30, femur 1.70–1.72/0.28, patella 1.41–1.45/0.30, chela (with pedicel) 2.46–2.61/0.54–0.58, chela (without pedicel) 2.34–2.50/0.54–0.58, hand length (without pedicel) 1.03–1.11, moveable finger length 1.24–1.41. Leg I: trochanter 0.31–0.34/0.22–0.23, femur 0.85–0.89/0.14–0.15, patella 0.57–0.59/0.13–0.15, tibia 0.62–0.66/0.11–0.12, basitarsus 0.42–0.45/0.09, telotarsus 0.46–0.5/0.09. Leg IV: trochanter 0.63–0.66/0.22–0.23, femur + patella 1.45–1.55/0.24–0.28, tibia 1.24–1.28/0.14–0.15, basitarsus 0.55–0.59/0.10–0.11, telotarsus 0.65–0.71/0.10.

#### Distribution.

China (Fujian).

#### Remarks.

*Stenohyahuangi* was described from a single female specimen by Hu and Zhang (2012). Although one of the original diagnostic characters is the presence of about 30 teeth on the movable chelal finger (Hu and Zhang 2012), we found a total of 47 teeth after inspection of the holotype. The most basal 14 teeth are rounded, while the other teeth have pointed tops (Fig. 11B). Herein, we describe more specimens of *S.huangi* from the type locality (Gushan Mountain in Fuzhou City, Fujian Province, China), including seven adult males, which allows the first description and illustrations of the male. Like some other species of *Stenohya*, *S.huangi* have sexually dimorphic pedipalps in which males have thinner pedipalps than females. Based on the holotype and the new specimens, we refine the diagnosis of *S.huangi*.

Males of *S.huangi* have fewer teeth on the movable chelal finger and very slender pedipalps without apophyses. They can be easily separated from the males of other *Stenohya* species with unarmed pedipalps, by the number of teeth on the movable chelal finger, and the proportions of pedipalpal femur and patella (Table 1).

**Table 1.** Numbers of teeth on movable chelal finger and proportions of pedipalpal femur and patella in male *Stenohya* species without apophyses on pedipalps.

Species	Numbers of teeth on movable chelal finger	Proportions of pedipalpal femur (length/breadth)	Proportions of pedipalpal patella (length/breadth)	References
*S.arcuata*	120–124	5.44–5.56	3.38–3.49	Guo et al. (2019)
*S.gruberi*	–	4.77	3.30	Ćurčić (1983)
*S.hainanensis*	93–100	5.00–5.52	3.44–3.71	Guo and Zhang (2016)
***S.huangi***	**30–33**	**7.64–7.96**	**6.32–6.45**	present paper
*S.kashmirensis*	70	4.90	2.60	Schawaller (1988)
*S.mahnerti*	90	4.40	3.20	Schawaller (1994)
*S.martensi*	87	6.70	5.60	Schawaller (1987)
*S.pengae*	45–47	6.79–7.20	6.17–6.25	Hu and Zhang (2012)
*S.setulosa*	76–89	5.15–5.19	3.28–3.39	Guo and Zhang (2016)
*S.tengchongensis*	92	4.14–4.43	2.40–2.75	Yang and Zhang (2013)
*S.xiningensis*	47	6.42	4.68	Zhao et al. (2011)

### ﻿Key to the genus *Stenohya* species from China

**Table:** 

1	Male pedipalpal femur and/or patella with projections on prolateral surfaces	**2**
–	Male pedipalpal femur and patella without prolateral projections	**4**
2	Male pedipalpal femur and patella with strong long peg-like projections on prolateral surfaces	***S.spinata* sp. nov.**
–	Male pedipalpal patella normal, femur with tubercles on prolateral face	**3**
3	Chelal hand with 14 tooth-shaped tubercles	***S.dongtianensis* Li & Shi, 2023**
–	Chelal hand with 42 tooth-shaped tubercles	***S.jiahensis* Li & Shi, 2023**
4	Male pedipalpal chelal hand with projection on prolateral surface	**5**
–	Male pedipalpal chelal hand without prolateral projection	**7**
5	Prolateral projection of male chelal hand with 2 hornlike bulges	***S.bicornuta* Guo, Zang & Zhang, 2019**
–	Prolateral projection of male chela hand with pointed projection	**6**
6	Male pedipalpal femur with a depression at the base of prolateral face; movable finger basally curved in ventral view	***S.curvata* Zhao, Zhang & Jia, 2011**
–	Male pedipalpal with straight femur; movable finger straight or slightly procurved	***S.meiacantha* Yang & Zhang, 2013**
7	Male pedipalpal femur strongly procurved	**8**
–	Male pedipalpal femur straight or slightly procurved	**9**
8	Male apex of pedipalpal coxa only with 4 long setae, short acicular seta absent	***S.arcuata* Guo, Zang & Zhang, 2019**
–	Male apex of pedipalpal coxa with 3 long setae and 10–12 short acicular ones	***S.setulosa* Guo & Zhang, 2016**
9	Each of chelal fingers with more than 85 teeth	**10**
–	Each of chelal fingers with less than 85 teeth	**11**
10	Male pedipalpal femur distally thickened, noticeably thicker than the basal section	***S.tengchongensis* Yang & Zhang, 2013**
–	Male pedipalpal femur not distally thickened	***S.hainanensis* Guo & Zhang, 2016**
11	Pedipalpal patella 4.00–6.00 times longer than broad	**12**
–	Pedipalpal patella 2.50–3.00 times longer than broad	***S.bomica* Zhao & Zhang, 2011**
12	Carapace with more than 30 setae	**13**
–	Carapace with less than 30 setae	***S.xiningensis* Zhao, Zhang & Jia, 2011**
13	Movable chelal finger with less than 50 teeth; galea divided into 4 or 5 branches	***S.huangi* Hu & Zhang, 2012**
–	Movable chelal finger with more than 50 teeth; galea divided into 6 branches	***S.pengae* Hu & Zhang, 2012**

## ﻿Discussion

Until now, seven of 23 *Stenohya* species have been recorded as having peculiar apophyses on the male pedipalps. *Stenohyaspinata* is the most exaggerated one, with various apophyses on the pedipalpal femur, patella, and chelal hand, while apophyses are absent on the pedipalpal patella in the other six species. We propose three hypotheses to explore the function of these apophyses on male pedipalps.

Hypothesis 1: The apophyses are helpful for holding the female’s pedipalps during mating. In some cheliferoid pseudoscorpions, sperm transfer is achieved by mating dances with bodily contact (Weygoldt 1966a, 1969). Mating commences when the male grasps the female’s pedipalp and ends when the spermatophore is transferred (Palen-Pietri et al. 2019). If these *Stenohya* pseudoscorpions have mating behavior that involves direct male–female contact, the apophyses on the male pedipalps may be used to increase the contact area, thus being helpful when holding the female’s pedipalps during mating. However, neobisiid pseudoscorpions have not been reported to have direct mating behavior, and there is no contact between male and female during sperm transfer (Weygoldt 1966a, 1969; Zeh and Zeh 1997). Furthermore, in these five *Stenohya* species, females lack special morphological structures to correspond to the modified male pedipalps, like a mortise and tenon structure that allows interaction between the two.

Hypothesis 2: The exaggerated male pedipalp is used to attract females for copulation. The pedipalp is the most important sensory organ in pseudoscorpions (Stemme and Pfeffer 2021), and it likely plays an important role in intraspecific communication. The exaggerated pedipalp of these *Stenohya* pseudoscorpions may be used for courtship display or bodily contact during mating. It should be noted that this hypothesis is also based on the presence of mating behavior in these species. In addition, mechanoreception may well be more useful than photoreception for pseudoscorpions (Weygoldt 1969; Tizo-Pedroso and Del-Claro 2008). Males are more likely to attract females for copulation using their pedipalps to make bodily contact with females if this hypothesis is true.

Hypothesis 3: The armed pedipalp is used as a weapon to fight with conspecific males. Animal weapons are very diverse structures, exhibiting different sizes and shapes within and between species (Emlen 2008; Rico-Guevara and Hurme 2019). Dimorphism ultimately results from differential selection acting on traits that have sex-dependent benefits and costs, leading the same trait toward different optima in each sex (Boisseau et al. 2020). The pedipalpal chela is a pseudoscorpions most effective defensive weapon, and males often use their pedipalpal chelae and fingers to fight with each other (Weygoldt 1966b). The apophyses on the pedipalps of these *Stenohya* pseudoscorpions may be helpful in fighting between intraspecific males during territorial defense or vying for mating opportunity. It is worth noting that males with larger chela have an advantage in the transfer of spermatophores, which can be attributed to their increased likelihood of interrupting mating and replacing smaller males under high-density conditions (Zeh 1987).

The Neobisiidae family is identified as having a reproductive strategy based on non-pairing sperm-transfer behavior, which may result in the lack of dancing behavior within this group (Weygoldt 1966a, 1969; Zeh and Zeh 1997). This would provide further support for hypothesis three. Nevertheless, there remains a significant gap in knowledge for the majority of pseudoscorpion species. In the absence of actual observations, each of these hypotheses discussed above seems to provide supporting and opposing evidence at the same time. Therefore, we believe that a key effort for future work should be focused on investigating the life histories of these *Stenohya* species that display sexual dimorphism. Ideally, the developmental evolution of sexual dimorphism should be inferred to in combination with their phylogenetic relationships. Future research efforts should also exploit recent advances in the fields of morphometrics, statistics, bioinformatics, and biomechanics. A more comprehensive and deep understanding of structure–function relationships in sexual dimorphism will provide better insight into the underlying evolutionary drivers of pseudoscorpion sexual dimorphism.

## Supplementary Material

XML Treatment for Stenohya spinata

XML Treatment for Stenohya huangi

## ﻿Additional information

### Author ORCIDs

Nana Zhan https://orcid.org/0009-0008-0554-9082

Zegang Feng https://orcid.org/0000-0002-5809-5984

Xiangbo Guo https://orcid.org/0000-0002-7074-8642

Feng Zhang https://orcid.org/0000-0002-3347-1031

### Data availability

All of the data that support the findings of this study are available in the main text or Supplementary Information.
